# Evaluation of an Automated High-Throughput Liquid-Based RNA Extraction Platform on Pooled Nasopharyngeal or Saliva Specimens for SARS-CoV-2 RT-PCR

**DOI:** 10.3390/v13040615

**Published:** 2021-04-02

**Authors:** Allen Wing-Ho Chu, Cyril Chik-Yan Yip, Wan-Mui Chan, Anthony Chin-Ki Ng, Dream Lok-Sze Chan, Ryan Ho-Ping Siu, Cheuk Yiu Tenny Chung, Jessica Pui-Ling Ng, Harsha Kittur, Garrett Lee Mosley, Rosana Wing-Shan Poon, Ricky Yin-To Chiu, Kelvin Kai-Wang To

**Affiliations:** 1 State Key Laboratory for Emerging Infectious Diseases, Carol Yu Centre for Infection, Department of Microbiology, Li Ka Shing Faculty of Medicine, The University of Hong Kong, Pok Fu Lam, Hong Kong; awhchu@hku.hk (A.W.-H.C.); mbally@hku.hk (W.-M.C.); anthonyng912@gmail.com (A.C.-K.N.); 2 Department of Microbiology, Queen Mary Hospital, Pok Fu Lam, Hong Kong; yipcyril@hku.hk (C.C.-Y.Y.); rosana@hku.hk (R.W.-S.P.); 3 PHASE Scientific International Limited, Kowloon, Hong Kong; dreamchan1220@gmail.com (D.L.-S.C.); ryan.siu@phasesci.com (R.H.-P.S.); tenny.chung@phasesci.com (C.Y.T.C.); jessica.ng@phasesci.com (J.P.-L.N.); harsha.kittur@phasesci.com (H.K.); garrett.mosley@phasesci.com (G.L.M.); ricky.chiu@phasesci.com (R.Y.-T.C.)

**Keywords:** COVID-19, SARS-CoV-2, liquid-based nucleic acid extraction, pooled specimens, mass screening, diagnostics, RT-PCR, nasopharyngeal swab, saliva

## Abstract

SARS-CoV-2 RT-PCR with pooled specimens has been implemented during the COVID-19 pandemic as a cost- and manpower-saving strategy for large-scale testing. However, there is a paucity of data on the efficiency of different nucleic acid extraction platforms on pooled specimens. This study compared a novel automated high-throughput liquid-based RNA extraction (LRE) platform (PHASIFY™) with a widely used magnetic bead-based total nucleic acid extraction (MBTE) platform (NucliSENS^®^ easyMAG^®^). A total of 60 pools of nasopharyngeal swab and 60 pools of posterior oropharyngeal saliva specimens, each consisting of 1 SARS-CoV-2 positive and 9 SARS-CoV-2 negative specimens, were included for the comparison. Real-time RT-PCR targeting the SARS-CoV-2 RdRp/Hel gene was performed, and GAPDH RT-PCR was used to detect RT-PCR inhibitors. No significant differences were observed in the Ct values and overall RT-PCR positive rates between LRE and MBTE platforms (92.5% (111/120] vs. 90% (108/120]), but there was a slightly higher positive rate for LRE (88.3% (53/60]) than MBTE (81.7% (49/60]) among pooled saliva. The automated LRE method is comparable to a standard MBTE method for the detection of SAR-CoV-2 in pooled specimens, providing a suitable alternative automated extraction platform. Furthermore, LRE may be better suited for pooled saliva specimens due to more efficient removal of RT-PCR inhibitors.

## 1. Introduction

Severe acute respiratory syndrome coronavirus 2 (SARS-CoV-2) has continued to spread globally despite implementation of various public health measures. Rapid resurgence of cases occurred after partial relaxation of social distancing measures [1]. However, aggressive diagnostic testing, together with prompt isolation and quarantine of close contacts, have successfully prevented further spread of the infection in some places [2,3].

SARS-CoV-2 has a positive-sense single-stranded RNA genome, which consists of two flanking untranslated regions, an orf1a/b gene encoding 16 non-structural proteins (nsp1–nsp16), several structural protein genes including spike (S), envelope (E), membrane (M) and nucleocapsid (N), and a number of accessory protein genes [4]. Reverse transcription-polymerase chain reaction (RT-PCR) is currently the gold standard for laboratory confirmation of SARS-CoV-2. Several gene regions have been evaluated as the target for RT-PCR, including the N, E, S, ORF1ab, nsp1 and nsp2 regions [5,6,7,8,9]. However, novel SARS-CoV-2 variants may contain mutations at the primer sites which evade detection, such as the failure of the S gene RT-PCR in the detection of the UK variant B.1.1.7 lineage [10].

During a typical SARS-CoV-2 RT-PCR-based diagnostic workflow, viral total nucleic acid (TNA) or RNA is extracted and purified using solid phase capture-and-release methods with magnetic beads or spin columns [11]. In these strategies, the ionic strength is increased to selectively bind nucleic acid to solid silica substrates, and later decreased to elute the RNA into a purified solution. While these approaches are widely used, they have several technical limitations. First, contaminants in the sample may interfere with the binding between nucleic acid and silica [12]. Second, the silica surface area available for nucleic acid binding is limited. Third, increased silica surface area requires more elution buffer, effectively diluting the nucleic acid in the final eluate [13]. Fourth, sample input volume is restricted due to limitations in column matrix capacity or magnetic bead strength.

In addition to these limitations, there are two major disadvantages associated with the use of bead or column-based extraction methods. First, bead or column-based methods are relatively expensive. Second, there has been a global shortage of these extraction kits during the COVID-19 pandemic. We and others have evaluated different low-cost and technically simple extraction methods, but the RT-PCR detection rate was found to be inferior to those extracted from a magnetic bead-based method [14,15].

Recently, a liquid-based RNA extraction (LRE) platform has been developed for viral RNA extraction. RNA can be extracted, purified, and concentrated without the need of silica beads or columns. Since the LRE method can concentrate the extracted RNA, it is especially suited for relatively large volume specimens.

Pooled specimens have now been used widely during the COVID-19 pandemic due to the large number of specimens to be tested [16,17,18]. Though promising, direct comparison of the LRE method with bead-based methods has not been evaluated on pooled specimens. In this study, we compared the detection rates of SARS-CoV-2 RT-PCR using an automated LRE platform against a magnetic-bead based extraction (MBTE) platform. We specifically assessed nasopharyngeal swab and saliva specimens since both specimen types are now used for pooled testing [11,19].

## 2. Materials and Methods

### 2.1. Individual Patient’s Specimens

Archived nasopharyngeal swab (NPS) or posterior oropharyngeal saliva specimens previously tested for SARS-CoV-2 were retrieved. These individual specimens were extracted using NucliSENS easyMAG extraction system (BioMérieux, Marcy-l′Étoile, France) and tested for SARS-CoV-2 by real-time RT-PCR targeting the RdRp/Hel gene as we described previously [6]. NPS and saliva were collected between 25 March 2020 and 27 November 2020, and were stored at −80 °C.

NPS were collected in 2 mL of viral transport medium. Posterior oropharyngeal saliva was collected by instructing the patient to spit into a sputum bottle and viral transport medium was then added, as we described previously [20,21]. The study was approved by the Institutional Review Board of The University of Hong Kong/Hospital Authority Hong Kong West Cluster (UW 20–286).

### 2.2. Pooling of Specimens

For 10-sample NPS pools, each SARS-CoV-2-positive NPS specimen was mixed with 9 SARS-CoV-2-negative NPS specimens in equal volume. For 10-sample saliva pools, each SARS-CoV-2-positive saliva specimen was mixed with 9 SARS-CoV-2-negative saliva specimens. As negative controls, we generated NPS or saliva pools consisting only of SARS-CoV-2 negative specimens. Then, 400 μL of each pool was subjected to RNA extraction by LRE or MBTE method.

### 2.3. Nucleic Acid Extraction of Pooled NPS or Saliva Specimens

#### 2.3.1. Magnetic Bead-Based Extraction (MBTE) Method

Total nucleic acid (TNA) extraction was performed using NucliSENS easyMAG extraction system (BioMérieux, Marcy-l′Étoile, France) according to the manufacturer’s protocol [22]. Briefly, 400 μL of each pooled sample was added to 2 mL of lysis buffer, vortexed briefly and incubate at room temperature for 10 min prior loading into the automation machine for extraction. After extraction, TNA was recovered using 25 μL, the lowest recommended volume for elution in the manufacturer’s instruction.

#### 2.3.2. Automated Liquid-Based Extraction (LRE) Method

**Figure 1:**
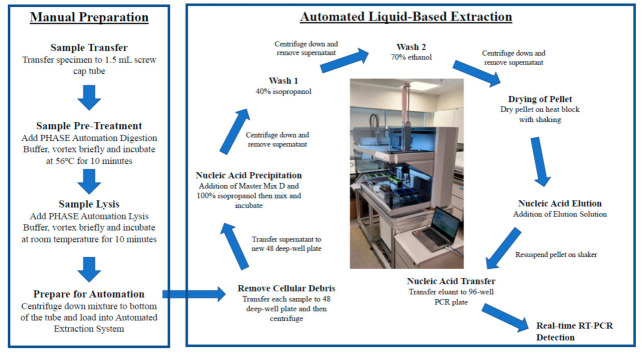
Workflow of the automated high-throughput liquid-based RNA extraction.

RNA extraction was performed using PHASIFY™ VIRAL RNA Extraction Kit (PHASE Scientific International Ltd., Hong Kong) in an automated high-throughput system (Tecan Evo 150 automated liquid handler) (Tecan Group Limited, Männedorf, Switzerland) (Figure 1). In brief, 40 μL of PHASE Automation Digestion Buffer was added to 400 μL of each pooled sample, vortexed briefly and allowed to incubate at 56 °C for 10 min. After incubation, 460 μL of PHASE Automation Lysis Buffer was added to the mixture and incubate for 10 min at room temperature prior loading into the automated machine for extraction. The elution volume was 25 μL.

### 2.4. Real-Time Reverse-Transcription Polymerase Chain Reaction

Real-time RT-PCR targeting the SARS-CoV-2 RdRp/Hel genes was performed as we described previously [6,21]. Briefly, each 20 μL reaction mixture contained 10 μL of 2× QuantiNova Probe RT-PCR Master Mix (Qiagen, Hilden, Germany), 0.2 μL of QN Probe RT-Mix, 1.6 μL of each 10 μM forward and reverse primer, 0.4 μL of 10 μM probe, 1.2 μL of nuclease-free water and 5 μL of TNA as the template. The thermal cycling condition was 45 °C for 10 min, 95 °C for 5 min, followed by 45 cycles of 95 °C for 5 s and 55 °C for 30 s.

Real-time PCR targeting the glyceraldehyde 3-phosphate dehydrogenase (GAPDH) gene was performed using PrimeScript™ RT Master Mix (Perfect Real Time) (Takara Bio Inc., Shiga, Japan). Briefly, the extracted RNA was first reverse transcribed to cDNA using the PrimeScript RT Master Mix (Takara Bio Inc., Kusatsu, Shiga, Japan). Each 10 μL reverse transcription reaction mixture contained 2 μL of 5× PrimeScript RT Master Mix (Perfect Real Time), 3 μL nuclease-free water and 5 μL of RNA as template. The thermal cycling condition was 37 °C for 15 min, 85 °C for 5 s and then held at 4 °C. For real-time PCR, each 10 μL reaction mixture contained 5 μL of TB Green Premix Ex Taq (Tli RNaseH Plus) (2×) (Takara Bio Inc., Kusatsu, Shiga, Japan), 0.3 μL of each 10 μM forward and reverse primer, 2.4 μL nuclease-free water, and 2 μL of cDNA as the template. The thermal cycling condition was 95 °C for 30 s, followed by 40 cycles of 95 °C for 5 s and 60 °C for 30 s.

### 2.5. Statistical Analysis

Statistical analysis was performed using SPSS v26.0, GraphPad PRISM v9.0.0 or GraphPad QuickCalcs. The Ct values were compared using one-way ANOVA with Dunn’s multiple comparison test. The detection rate of LRE and MBTE pools were compared using McNemar’s test.

## 3. Results

We included 120 positive pools, with each containing one positive specimen and 9 negative specimens. Sixty were NPS pools and 60 were saliva pools. The median Ct values of the positive individual specimens in each pool was 27.3 (interquartile range 22.6–29.8) and 28.0 (IQR 25.5–29.3) for NPS and saliva, respectively.

The overall positive rate was 92.5% (111/120) and 90% (108/120) for LRE and MBTE, respectively (Table 1), with no statistically significant difference (*p* = 0.629). All NPS pools tested positive by either LRE or MBTE. For saliva pools, 2 tested negative by either LRE or MBTE. The overall positive percent agreement (PPA) was 85.6% (101/118) (95% CI: 78.1–90.8%). As negative controls, we included 10 NPS and 4 saliva pools, each consisting of 10 negative specimens. All 14 negative pools tested negative for SARS-CoV-2.

Next, we performed subgroup analysis of different specimen types. For NPS, SARS-CoV-2 could be detected in all pools with either LRE or MBTE, where 95% (57/60) were detected for both extraction methods (Table 2). The NPS positive rates were similar between LRE (96.7% [58/60]) and MBTE (98.3% [59/60]) (*p* = 1.000) (Table 1). For saliva specimens, SARS-CoV-2 was detected in 96.7% (58/60) with either LRE or MBTE, but only 73.3% (44/60) were detected with both extraction methods. The saliva positive rate was higher for LRE (88.3% [53/60]) than that of MBTE (81.7% [49/60]), though not reaching statistical significance (*p* = 0.424).

There was no significant difference in the positive rates for LRE between NPS and saliva in pooled specimens (96.7% [58/60] vs. 88.3% [53/60], *p* = 0.163). The PPA of NPS (95% [57/60; 95% CI: 86.3–98.6%]) was significantly higher than that of saliva (75.9% [44/58; 95% CI: 63.5–85.0%]) (*p* = 0.0036).

**Figure 2:**
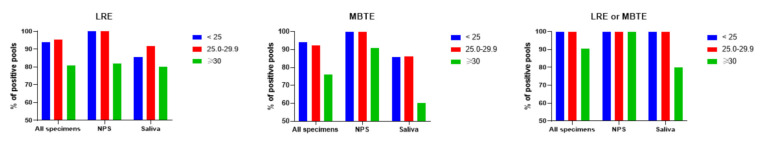
Comparison of SARS-CoV-2 RT-PCR positive rates between different Ct values. Different color bars indicate different Ct values. Abbreviations: LRE, liquid-based RNA extraction; MBTE, magnetic bead-based total nucleic acid extraction; NPS, nasopharyngeal swab.

To identify the reason for the failure to detect SARS-CoV-2 among pooled specimens, we assessed the positive rates based on different Ct value ranges (Figure 2). For both LRE and MBTE, the detection rates were lowest when the Ct value was 30 or above irrespective of specimen types.

To determine whether the SARS-CoV-2 negative results were due to RT-PCR inhibitors, we performed real-time RT-PCR for GAPDH on the RNA or TNA extracts that were RT-PCR negative for SARS-CoV-2 (9 with LRE platform; 12 with MBTE platform). For NPS specimens, GAPDH was detected in all 3 specimens with discrepant results, suggesting the absence of RT-PCR inhibitors in the nucleic acid extracted by either LRE or MBTE platform. However, for saliva specimens, GAPDH was only successfully detected in all RNA extracts from the LRE platform only. For the TNA extracted from the MBTE platforms, GAPDH could be detected in only 63.6% (7/11), suggesting the presence of RT-PCR inhibitors in 36.4% of these TNA (Table 2).

**Figure 3:**
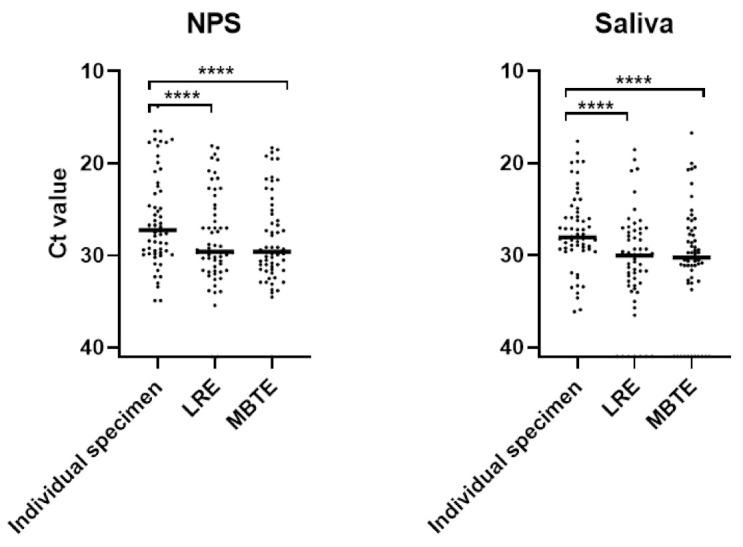
Comparison of the Ct values in SARS-CoV-2 real-time RT-PCR, **** *p* < 0.0001. Abbreviations: LRE, liquid-based RNA extraction; MBTE, magnetic bead-based total nucleic acid extraction; NPS, nasopharyngeal swab.

For NPS specimens, both LRE (median Ct 29.6, IQR: 25.0–31.6) and MBTE (median Ct 29.6, IQR: 25.2–31.5) pools had significantly higher Ct values than individual specimens (median Ct 27.3, IQR 22.6–29.8) (adjusted *p* < 0.0001), but there was no significant difference between LRE and MBTE pools (adjusted *p* = 0.82) (Figure 3). Similarly, for saliva specimens, both LRE (median Ct: 30.0, IQR: 27.5–33.2) and MBTE (median Ct: 30.2; IQR: 27.4–32.8) pools had significantly higher Ct values than individual specimen (median Ct: 28.0; IQR: 25.5–29.3) (adjusted *p* value < 0.0001), but there was also no difference between LRE and MBTE pools (adjusted *p* = 1.0).

## 4. Discussion

### 4.1. Summary of Principle Findings

This study evaluated the detection rate of SARS-CoV-2 using a novel automated high-throughput LRE. There was no significant difference in the positive rates and Ct values in SARS-CoV-2 RT-PCR for pooled NPS or saliva specimens extracted using the LRE and MBTE methods. For saliva pools, there was a trend towards higher detection rate with the LRE method than that of MBTE method. Analysis with the housekeeping gene GAPDH showed the presence of RT-PCR inhibitors in the TNA extracted using the MBTE method in 36.4% of the specimens that failed detection of SARS-CoV-2. Our results suggest that LRE is comparable to MBTE for pooled specimens and LRE may be superior to MBTE for saliva specimens.

### 4.2. Comparison with Other Studies

Most studies on pooled testing assess nasopharyngeal or oropharyngeal specimens [17]. However, saliva has been used during the COVID-19 pandemic because of the high sensitivity and the ease of specimen collection [20,21,23]. Barat et al. has evaluated pooled saliva testing using a MBTE platform for TNA extraction [11]. In our current study, we showed that the benefit of the LRE was most notable for pooled saliva specimens, in which the positive rate was higher for LRE than that of MBTE (88.3% vs. 81.7%). Further analysis showed that the lower positive rate for MBTE was due to the presence of RT-PCR inhibitors, which were detected in 36.4% of MBTE specimens that tested negative for SARS-CoV-2. Moreover, RT or PCR inhibitors were not found in any LRE-extracted saliva specimens. The presence of inhibitors in pooled specimen extracted from MBTE is of concern, as many commercial assays do not include an internal control for RT-PCR inhibitors, and therefore may lead to false negative results. Ambers et al. showed that saliva can reduce the signal in real-time PCR reactions, although the exact constituent that affects PCR amplification is not known [24].

The PPA between LRE and MBE was significantly lower for saliva than that of NPS. Excluding the 4 saliva pools with RT-PCR inhibitors detected, 10 of 56 pools have discrepant results between LRE and MBTE. Our results suggest that RNA extraction from pooled saliva specimens require further protocol refinement. Several components of saliva can affect nucleic acid extraction. First, mucus plays an important role in host defense by trapping respiratory pathogens. However, the viscous mucus layer can also protect the virus from the actions of the lysis buffer [25]. Second, the presence of ribonucleases in saliva can degrade RNA, reducing the amount of RNA to be detected in subsequent RT-qPCR assays [26]. Third, proteases in saliva could disrupt the viral structure, allowing ribonucleases to degrade viral genome RNA [27,28]. The improved elimination of the RT-PCR inhibitors in LRE method may be due to the use of proteinase K to digest saliva proteins and to reduce the viscosity, along with the removal of saliva matrix and cellular debris during the precipitation process.

In this study, the LRE platform is an automated high-throughput system which can process up to 96 samples simultaneously. This is a major advantage over column-based extraction method which can only be performed manually. Automation is important for large scale testing. First, the hands-on-time for the automated LRE extraction is only about 20 min, greatly reducing the manpower cost. Second, automation would reduce the chance of human error during sample processing and minimize the infectious risk to the technicians.

Due to the effect of dilution, the detection rate decreases if more samples are included in a single pool [17,19,29,30]. However, pooling fewer specimens would incur a higher cost. Our study chose a pooling strategy of 10 samples as it provides a balance between practicality in reducing workload and the ease of identifying positive samples within the pool without overly sacrificing on the sensitivity of the real-time RT-PCR test.

In view of the shortage of bead-based or column-based extraction kits, alternative extraction methods have been proposed [14,31]. However, all had lower extraction efficiency than bead or column-based extraction methods. In our current study, the LRE method had similar performance as a widely used commercial kit. Hence, LRE is a suitable alternative without sacrificing the detection rate.

### 4.3. Limitations of This Study

There are several limitations in this study. First, the NPS and saliva are not paired specimens collected from same individuals at the same time, we could not directly compare the results between NPS and saliva specimens for individual patients. Second, since we used archived specimens, the viral load may have been reduced from the original specimen due to storage. However, this would not affect the comparison between the LRE and MBTE platforms. To achieve a fair comparison between the two platforms, the input and elution volume was the same for both platforms.

### 4.4. Conclusions and Implications for Clinical Practice and Research Studies

During the COVID-19 pandemic, large scale testing for SARS-CoV-2 has been implemented as one of the public health measures to identify patients for early treatment and isolation to prevent further transmission [3]. Because the current diagnostic capacity cannot cope with the large number of specimens, pooled testing has been advocated [11,19]. This study showed that the LRE automated system has similar performance for NPS and better performance for saliva than MBTE in pooled specimens. The combination of the novel extraction system and pooling strategy can greatly reduce the number of extraction and qPCR kits required whilst increasing the testing capacity. As this novel method does not require the need of magnetic beads or silica-based columns, the kit is more readily available during the time where reagent kits are in high demand. Furthermore, LRE systems do not have components that require sensitive or expensive production, such as magnetic beads or column matrices, resulting in a more cost-efficient extraction product. LRE is particularly suited for pooled testing because it can accommodate a larger input volume that is otherwise not recommended for column-based methods. Our study also highlighted the importance of checking RT-PCR inhibitors in pooled saliva specimens as it may produce false negative results. This is especially important as saliva has been proposed to be a potential specimen for large scale testing [32].

## Figures and Tables

**Table 1:** Comparison between the detection rate in 120 positive pooled respiratory specimens extracted by liquid-based RNA extraction and magnetic bead-based total nucleic acid extraction platforms.

Specimen Type	No. Positive (%)		*p*-Value ^a^
	LRE	MBTE	
NPS (*n* = 60)	58 (96.7) ^b^	59 (98.3) ^b^	1.000
Saliva (*n* = 60) ^b^	53 (88.3) ^b^	49 (81.7) ^c^	0.424
Total (*n* = 120)	111 (92.5)	108 (90.0)	0.629

Abbreviations: LRE, liquid-based RNA extraction; MBTE, magnetic bead-based total nucleic acid extraction; NPS, nasopharyngeal swab. ^a^ By McNemar’s test; ^b^ GAPDH could be detected in all pools which tested negative for SARS-CoV-2; ^c^ GAPDH could not be detected in 4 of 11 pools which tested negative for SARS-CoV-2.

**Table 2:** Pooled samples that tested negative for SARS-CoV-2 in at least one extraction method.

SARS-CoV-2 RT-PCR	No. of Pools (%)	No. of Pools with GAPDH Detected by RT-PCR
**NPS (*n* = 60)**		
LRE positive MBTE positive	57 (95)	Not tested
LRE positive MBTE negative	1 (1.7)	1 ^a^
LRE negative MBTE positive	2 (3.3)	2 ^b^
**Saliva (*n* = 60)**		
LRE positive MBTE positive	44 (73.3)	Not tested
LRE positive MBTE negative	9 (15)	5 ^a^
LRE negative MBTE positive	5 (8.3)	5 ^b^
LRE negative MBTE negative	2 (3.3)	2 ^c^

Abbreviations: LRE, liquid-based RNA extraction; MBTE, magnetic bead-based total nucleic acid extraction; ^a^ From LRE platform; ^b^ From MBTE platform; ^c^ From both LRE and MBTE platform.

## Data Availability

The data presented in this study are available on request from the corresponding author.
